# Association of HMGCR polymorphism with late-onset Alzheimer's disease in Han Chinese

**DOI:** 10.18632/oncotarget.8176

**Published:** 2016-03-18

**Authors:** Xiao-Long Chang, Lin Tan, Meng-Shan Tan, Hui-Fu Wang, Chen-Chen Tan, Wei Zhang, Zhan-Jie Zheng, Ling-Li Kong, Zi-Xuan Wang, Teng Jiang, Jin-Tai Yu, Lan Tan

**Affiliations:** ^1^ Department of Neurology, Qingdao Municipal Hospital, Dalian Medical University, Qingdao, PR China; ^2^ College of Medicine and Pharmaceutics, Ocean University of China, Qingdao, PR China; ^3^ Department of Neurology, Qingdao Municipal Hospital, School of Medicine, Qingdao University, Qingdao, PR China; ^4^ Department of Neurology, Qingdao Municipal Hospital, Nanjing Medical University, Qingdao, PR China; ^5^ Department of Geriatric, Qingdao Mental Health Center, Qingdao, PR China; ^6^ Department of Neurology, Nanjing First Hospital, Nanjing Medical University, Nanjing, PR China

**Keywords:** Alzheimer's disease, HMGCR, rs3846662, polymorphism

## Abstract

The 3-hydroxy-3-methylglutaryl-CoA reductase (*HMGCR*) acts as a potential genetic modifier for Alzheimer's disease (AD). Previous reports identified that *HMGCR* rs3846662 polymorphism is associated with biosynthesis of cholesterol in AD pathology. In order to assess the involvement of the *HMGCR* polymorphism in the risk of late-onset AD (LOAD) in northern Han Chinese, we performed a case–control study of 2334 unrelated subjects (984 cases and 1350 age- and gender-matched controls) to evaluate the genotype and allele distributions of the *HMGCR* rs3846662 with LOAD. The genotype distribution (GG, AG, AA) of rs3846662 was significantly different between LOAD patients and controls (*P* = 0.003), but the allele distribution did not reach a significant difference (*P* = 0.614). After adjusting for age, gender and the *APOE* ε4 status, the minor A allele of rs3846662 was validated as a protective factor for LOAD in dominant model (OR = 0.796, *P* = 0.02, 95% CI = 0.657–0.965). Interestingly, we observed rs3846662 polymorphism was only significantly associated with LOAD in *APOE* ε4 non-carriers (OR = 0.735, *P* = 0.005, 95% CI = [0.593, 0.912]). In conclusion, our study demonstrates A allele of *HMGCR* rs3846662 acts as a protective factor for LOAD in northern Han Chinese.

## INTRODUCTION

Alzheimer's disease (AD), as the most common type of dementia for the aged people, is a multifactorial disease with complex etiology over genetic and environmental factors [1, 2]. The late-onset AD (LOAD) is identified as the most common form and has a complex genetic issue. Until now, only the ε4 allele of apolipoprotein E (*APOE*) gene has been identified unequivocally as a major genetic factor to LOAD. However, *APOE* alone can only explain less than 50% of the genetic component of LOAD [3]. Recent several large genome-wide association studies (GWAS) based on Caucasians have identified numbers of several risk genes that may affect LOAD susceptibility [4, 5]. However, these GWAS-linked loci still could not fully account for the genetic factor of LOAD, indicating that there are additional risk loci for LOAD remaining to be discovered [6].

3-hydroxy-3-methylglutaryl-CoA reductase (HMGCR) encodes part of the statin-binding domain of the enzyme, which serves as the rate-limiting step in cholesterol synthesis [7, 8]. Cholesterol modulates alpha-secretase cleavage of amyloid precursor protein and generation of amyloidogenic Aβ peptides [9, 10]. Since hypercholesterolemia accelerates amyloid pathology [11], cholesterol depletion inhibits the generation of beta amyloid in hippocampal neurons [12], a direct correlation has also been suggested. Furthermore, individuals with elevated blood cholesterol were more vulnerable to AD and AD patients shown higher serum levels of total and low density lipoprotein (LDL) cholesterol compared to cognitive intact people [13]. In addition, previous studies have focused on *HMGCR* polymorphism modifying the risk to LOAD [14–16]. Recently, V Leduc et al. performed a study in a founder population and in two distinct mixed North American populations and found that the intron 13 SNP rs3846662 A allele were shown to be strongly protective for LOAD [15]. As rs3846662 is an expression Quantitative Trait Loci (eQTL) and is able to alter the binding motif to regulate HMGCR exon 13 skipping [17], and the A allele at rs3846662 increased proportion of *HMGCR* mRNA [18–21]. All above evidences unequivocally make rs3846662 as an important susceptibility locus for LOAD.

To date, the association between rs3846662 polymorphism with AD was exclusively conducted in Caucasians and no related studies have been conducted in Asian population. Given the potential important role of rs3846662 polymorphism in LOAD, additional independent replications are necessary in other races such as northern Han Chinese, as variations and their frequencies of *HMGCR* in various ethnic groups might be different. In the present study, we firstly conducted the genetic association study between rs3846662 and LOAD in a Northern Han Chinese population.

## RESULTS

The demographics of the study and the distribution of *APOE* allele (dichotomized into *APOE* ε4 carriers and *APOE* ε4 non-carriers) were summarized in Table 1. No significant differences were observed for age (*P* = 0.129) and gender (*P* = 0.069) between LOAD patients and controls. MMSE scores were significantly lower in patients than in control subjects (LOAD: 11.99 ± 6.20, control: 28.49 ± 1.09, *P* = 0.0001). The presence of the *APOE* ε4 significantly raised the risk of LOAD in cases compared with control subjects (*P* = 0.0001, OR = 2.413, 95% CI = 1.963 – 2.967).

**Table 1:** Characteristics of the study groups

Characteristics	AD patients (*n* = 984)	Control (*n* = 1350)	*P* value	OR (95% CI)
Age, mean + SD	75.15 + 6.1	75.48 + 6.5	0.219	
Gender, *n* (%)				
Male	406(41.3%)	608(45.0%)	0.069	
Female	578(58.7%)	742(55.0%)		
MMSE score, mean + SD	11.99 + 6.2	28.49 + 1.1	0.0001	
*ApoE* genotypes, *n* (%)				2.413 (1.963–2.967)^↑^
*ApoE* ε4(+)	280 (28.5%)	191 (14.1%)	0.0001	
*ApoE* ε4(−)	704 (71.5%)	1159 (85.9%)		

Abbreviations: AD, Alzheimer disease; OR, odds ratio; CI, confidence interval; SD, standard deviation; MMSE, Mini-Mental State Examination; *ApoE* ε4 (+), ε2/ε4, ε3/ε4, ε4/ε4; *ApoE* ε4 (−), ε2/ε2, ε2/ε3, ε3/ε3.

* *P* value was calculated with the age of onset for AD patients and age at examination for control subjects. Differences in age and MMSE score were measured by Student's *t* test. Differences in sex and genotype of *ApoE* were calculated by Pearson's χ^2^ test.

↑ OR value represents odd ratio for AD patients compared to control subjects.

Genotype distribution of rs3846662 was in Hardy–Weinberg equilibrium (HWE) in control subjects (*P* > 0.05). The genotype distribution (GG, AG, AA) of rs3846662 was significantly different between LOAD patients and controls (*P* = 0.003), but the allele distribution did not reach a significant difference (*P* = 0.614). Our sample had a 95% power to detect an OR of 0.80 for LOAD within a significance level of 0.05. (Table 2)

**Table 2:** Distribution of the rs3846662 genotypes and alleles in AD cases and controls stratified by ApoE ε4 status

		Genotypes *n* (%)				Allele *n* (%)		
rs3846662	*N*	GG	AG	AA	*P*	G	A	*P*
AD	984	268 (27.2)	470 (47.8)	246 (25.0)	0.003^*^	1006 (51.1)	962 (48.9)	0.614
Controls	1350	310 (23.0)	740 (54.8)	300 (22.2)		1360 (50.4)	1340 (49.6)	
*ApoE* ε4 (−)								
AD	704	198 (28.1)	340 (48.3)	166 (23.6)	0.006^*^	736 (52.3)	672 (47.7)	0.17
Controls	1159	259 (22.3)	640 (55.2)	260 (22.4)		1158 (50%)	1160 (50%)	
*ApoE* ε4 (+)								
AD	280	70 (25.0)	130 (46.4)	80 (28.6)	0.170	270 (48.2)	290 (51.8)	0.16
Controls	191	51 (26.7)	100 (52.4)	40 (20.9)		202 (52.9)	180 (47.1)	

Abbreviations: *P, P*-value calculated from Pearson's χ^2^ test.

* *P*, significant values.

In addition, we performed a multivariate logistic regression analysis adjusting for age, gender, and *APOE* to assess the effect of rs3846662 on LOAD risk in three genetic models. We found that the rs3846662 significantly protected LOAD in dominant model (OR = 0.796, *P* = 0.02, 95% CI = [0.657, 0.965]). No significant difference was observed in recessive and additive models. (Table 3)

**Table 3:** Logistic regression analysis of rs3846662 polymorphism in AD patients and controls

Model	Total sample^a^		*P for ApoE × SNP interaction*	*ApoE ε4 allele* (*–*)^b^		*ApoE ε4 allele* (*+*)^b^	
	OR (95% CI)	*P*		OR (95% CI)	*P*	OR (95% CI)	*P*
Dominant	0.796 (0.657–0.965)	0.020^*^	0.109	0.735 (0.593–0.912)	0.005^*^	1.080 (0.709–1.644)	0.721
Recessive	1.155 (0.949–1.406)	0.151	0.163	1.073 (0.859–1.340)	0.536	1.527 (0.988–2.361)	0.057
Additive	0.966 (0.856–1.089)	0.569	0.063	0.908 (0.793–1.041)	0.168	1.200 (0.926–1.553)	0.168

Abbreviations: *P, P*-value calculated from binary logistic regression; SNP: single nucleotide polymorphism; Dominant: dominant model; Recessive: recessive model; Additive: additive model.

a Adjusted for age, gender and *ApoE* ε4 status.

b Adjusted for age and gender.

* *P* < 0.05, significant values.

Furthermore, we tested the influence of interaction between rs3846662 and *APOE* genotype on the risk of LOAD in logistic regression models and we were unable to detect an interaction between *APOE* ε4 and rs3846662 in three genetic models. (Table 3) To further investigate whether the presence of the *APOE* ε4 allele modified significantly the association of rs3846662 with LOAD, the total group was stratified in by *APOE* ε4 status. After divided the subjects by *APOE* ε4 allele status, the discrepancy of genotype distribution of rs3846662 between cases and controls was more remarkable in the *APOE ε4* non-carriers (*P* = 0.006). However, this diversity didn't reach significant in *APOE ε4* allele carriers. Besides, we also repeat logistic regression analysis adjusting for age and gender in each subgroup with all the three genetic models. In *APOE* ε4 allele non-carriers, only dominant model revealed remarkable protective role in LOAD (OR = 0.735, *P* = 0.005, 95% CI = [0.593, 0.912]) while the other two models showed no significance.

## DISCUSSION

We firstly conducted the association study of *HMGCR* rs3846662 polymorphism with the risk of LOAD in Northern Han Chinese population. The minor A allele at rs3846662 was significantly decreased risk of LOAD. This discovery was further confirmed by logistic regression analysis adjusting for age, gender, and *APOE* ε4. Interestingly, after stratification by the status of *APOE* ε4, all these positive results remained only in the *APOE* ε4 non-carriers.

Our study was largely in accordance with previous reports [14–16, 21]. Two independent studies have been suggested that rs3846662 was a genetic modifier for risk of MCI conversion to AD as well as statin responsiveness [15, 21]. Our study confirmed the conclusion and highlighted the protective role of *HMGCR* in AD pathogenesis. Our results were mostly in accordance with Christopher R Simmons's and V Leduc's study [15, 21]. In Christopher's study, after stratification by *APOE ε4*, patients without G allele in rs3846662 increased risk in LOAD compared to G allele carriers (*P* = 0.003, OR = 1.141). Likewise, V Leduc's study was able to detect a significant effect of G allele negative genotype (AA) on the age of onset and exerted a delayed age of onset in women. It should be noted that only the A allele (AA+ AG) under dominant model significantly protected LOAD in our study. This discrepancy may be explained by genetic heterogeneity since no similar study has been carried out in Asian or Chinese population.

It seems that the *HMCGR* gene itself is capable of modify AD pathogenesis. Variants in *HMGCR* at rs3846662 directly modulated alternative splicing of *HMGCR* exon13 and the alternative spliced variant (exon13) could not restore *HMGCR* activity when expressed in *HMGCR* deficient UT-2 cells [18], thus this means variant rs3846662 may alter enzyme activity and/or statin sensitivity. Furthermore, the rs3846662 related variation produced an *HMGCR* isoform with reduced statin sensitivity compared with the full-length or classic isoforms. This isoform played a decisive role for low-density lipoprotein cholesterol and triglyceride in response to statin treatment among individuals [20]. However, the protective role of rs3846662 is mediated by brain cholesterol homeostasis or by direct modulation is still in debate. In another study, the presence of *HMGCR* (5′-UTR) polymorphism is crucial to the association between *ABCA1* polymorphism and AD [16]. In central nervous system, *APOE* serves as the major cholesterol carrier to neurons [7], and *ABCA1* regulates the secretion of APOE from astrocytes and microglia. These genes or proteins may have complementary roles in cholesterol homeostasis in central nervous system.

Unfortunately, our study did not find any association between *HMGCR* rs3846662 polymorphism and *APOE* ε4 carriers. The lack of association in *APOE* ε4 carriers may possibly due to a limited number of cases and controls of *APOE* ε4 carries. This study didn't have enough power to association of *HMGCR* polymorphism in such a small sub-cohort. Thus it should be validated in a larger cohort in the future. The other possible explanation is that the *HMGCR* no longer modulate AD pathology in *APOE* ε4 carriers, which might reflect the exhaustion of HMGCR system in end-stage AD [15]. It is difficult to interpret the biochemical consequences of a potential interaction between *HMGCR* and *APOE* due to the poor understanding of the mechanisms of brain cholesterol homeostasis. The weight for *HMGCR* polymorphism in disturbing cholesterol homeostasis is currently unclear. However, recent study suggested that minor A allele in rs3846662 was associated with a blunted response to statin therapy [20, 22]. This phenomenon may partly attribute to an increased proportion of *HMGCR* lacking exon 13 which encodes a portion of both the active site of the enzyme and the statin binding site. A allele of rs3846662 may be less likely to respond to statins in AD trials while rs3846662 G carriers are more likely to respond to statin therapy in AD [21, 23].

In conclusion, our study certificated that the *HMGCR* may act as a genetic modifier for LOAD risk and the minor A allele on rs3846662 is associated with a protective for LOAD in a Chinese Han population. The association needs to be replicated in large cohorts and in different ethnic populations, because the variations of heredity are diverse in different ethnic and geographical origin. Besides, the gene and cholesterol synthesis should be analyzed to evaluate the relationship between *HMGCR* and LOAD, in order to better explain the role of *HMGCR* polymorphism in the pathological process of AD.

## MATERIALS AND METHODS

### Subjects information

We investigated 984 sporadic LOAD patients (mean age at onset: 75.15 ± 6.1 years; male 406 and female 578) and 1350 healthy controls (mean age at examination: 75.48 ± 6.5 years; male 608 and female 742) matched for age and gender. All subjects were unrelated Northern Han Chinese residents originally from Shandong Province. The AD patients were consecutively enrolled from the Department of Neurology at Qingdao Municipal Hospital, the Affiliated Hospital of the Medical College of Qingdao University, and several other hospitals. The diagnosis of probable AD were implemented by at least 3 neurologists in accordance with the diagnostic criteria of the National Institute of Neurological and Communication Disorders and Stroke–Alzheimer Disease and Related Disorders Association (NINCDS-ADRDA) [24]. The information of patients, including age at onset and family history, were determined by their kinship members. The control subjects were restricted in those whom confirmed healthy and normal in medical history, general examination, neurological examination, laboratory examination, and Mini Mental State Examination (MMSE > 28) from the Health Examination Center of the Qingdao Municipal Hospital. Informed consent was obtained from each participant or their legal guardian and our study was approved by the institutional ethics committee of Qingdao Municipal Hospital.

### DNA extraction and genotyping

Genomic DNA was extracted from the peripheral blood sample (AD and control cases) using the Wizard genomic DNA purification kit (Cat. #1125, Promega, USA). The rs3846662 and *APOE* polymorphism was genotyped with a method of polymerase chain reaction-ligase detection named SNPscan^™^, a patent-pending technology which guaranteed more than 99% genotyping accuracy from the Shanghai Genesky Biotechnology Company. Data analysis was fulfilled by GeneMapper Software v4.1 (Applied Biosystems, USA). Randomly selected DNA samples from each genotype were analyzed in duplicates using ligation detection reaction (LDR) and sequence analysis.

### Statistical analysis

HWE version 1.20 (Columbia University, New York, NY) was used to exclude deviations from Hardy-Weinberg equilibrium for control subjects. Differences of the characteristics between AD patients and control subjects were calculated through the Student *t-*test or the chi-square test. Differences of genotype and allele frequencies were compared using chi-square test. OR value and the 95% confidence interval (CI) for assessing genotypic and allelic associations with AD were analyzed using logistic regression, adjusting for age of onset (age at examination for control subjects), gender, and *APOE* ε4 status (presence or absence of an ε4 allele) under various genetic models. The models were defined as 1 (AA + AG) versus 0 (GG) for dominant, 1 (AA) versus 0 (AG + GG) for recessive, and 0 (GG) versus 1 (AG) versus 2 (AA) for additive (G: major allele; A: minor allele). The significance of SNP × *APOE* interaction was also tested by logistic regression. The statistical power was calculated with STPLAN 4.3 software. Data were analyzed using SPSS 17.0 (SPSS Inc, Chicago, IL). The level of significance for statistic tests in present study was defined as *P <* 0.05.
